# Identification of Cytotoxic Drugs That Selectively Target Tumor Cells with MYC Overexpression

**DOI:** 10.1371/journal.pone.0027988

**Published:** 2011-11-23

**Authors:** Anna Frenzel, Hanna Zirath, Marina Vita, Ami Albihn, Marie Arsenian Henriksson

**Affiliations:** Department of Microbiology Tumor and Cell Biology (MTC), Karolinska Institutet, Stockholm, Sweden; Karolinska Institutet, Sweden

## Abstract

Expression of MYC is deregulated in a wide range of human cancers, and is often associated with aggressive disease and poorly differentiated tumor cells. Identification of compounds with selectivity for cells overexpressing MYC would hence be beneficial for the treatment of these tumors. For this purpose we used cell lines with conditional MYCN or c-MYC expression, to screen a library of 80 conventional cytotoxic compounds for their ability to reduce tumor cell viability and/or growth in a MYC dependent way. We found that 25% of the studied compounds induced apoptosis and/or inhibited proliferation in a MYC-specific manner. The activities of the majority of these were enhanced both by c-MYC or MYCN over-expression. Interestingly, these compounds were acting on distinct cellular targets, including microtubules (paclitaxel, podophyllotoxin, vinblastine) and topoisomerases (10-hydroxycamptothecin, camptothecin, daunorubicin, doxorubicin, etoposide) as well as DNA, RNA and protein synthesis and turnover (anisomycin, aphidicholin, gliotoxin, MG132, methotrexate, mitomycin C). Our data indicate that MYC overexpression sensitizes cells to disruption of specific pathways and that in most cases c-MYC and MYCN overexpression have similar effects on the responses to cytotoxic compounds. Treatment of the cells with topoisomerase I inhibitors led to down-regulation of MYC protein levels, while doxorubicin and the small molecule MYRA-A was found to disrupt MYC-Max interaction. We conclude that the MYC pathway is only targeted by a subset of conventional cytotoxic drugs currently used in the clinic. Elucidating the mechanisms underlying their specificity towards MYC may be of importance for optimizing treatment of tumors with MYC deregulation. Our data also underscores that MYC is an attractive target for novel therapies and that cellular screenings of chemical libraries can be a powerful tool for identifying compounds with a desired biological activity.

## Introduction

The MYC transcription factor is one of the most potent and commonly deregulated oncoproteins in human cancers [1], [2]. It is a member of the basic helix-loop-helix/leucine zipper (bHLH-Zip) family of transcriptional regulators, and requires heterodimerization with the Max protein for DNA binding to promote its biological functions [3], [4]. In addition to its role in cell transformation, MYC plays an important role in the regulation of cellular functions such as cell cycle progression, cell growth, apoptosis, proliferation, differentiation, angiogenesis and maintenance of genetic stability [2], [3], [5]. Moreover, the dual role of the MYC family members in regulation of the cell cycle and apoptosis implicate them as therapeutic targets [6], [7].

Deregulation of the MYC family members *c-MYC*, *MYCN* and *MYCL* are implicated in the genesis of human cancers and is frequently strongly correlated with poor prognosis [1], [2]. In Burkitt lymphoma (BL), *c-MYC* is translocated to one of the immunoglobulin loci resulting in its constitutive expression [8]. In many solid tumors, *MYC* is instead amplified or otherwise deregulated. For example, amplification of *MYCN* is a hallmark of neuroblastoma (NB), and is also observed in other tumors including medulloblastoma, rhabdomyosarcoma, glioma and lung cancer (for review see [1], [9]. Current treatment regimens for patients with BL includes a battery of cytotoxic drugs such as alkylating agents, microtubule-targeting agents, topoisomerase inhibitors and antibiotics together with the dihydrofolate reductase (DHFR) inhibitor methotrexate [10]–[12]. Despite documented efficacy of current treatment schemes for BL, these are often associated with undesired and sometimes severe side-effects [10], [12]. Treatment schemes for NB patients consist of multimodal therapy, combining surgery, radiotherapy and chemotherapy, complemented in some cases with bone marrow transplantation, depending on disease stage. Chemotherapeutic treatment mainly involves combinations of alkylating agents, microtubule-targeting agents, topoisomerase inhibitors, and antibiotics. *MYCN*-amplification is associated with the most aggressive phenotype of neuroblastoma. The survival rate for high-risk NB is approximately 30% despite advances in treatment strategies. Typically neuroblastomas are initially sensitive to cytotoxic agents, but drug resistance often develops in high-risk disease. Current efforts are trying to avoid or overcome this effect [13]–[16]. Thus, identification of novel non cross-resistant therapies and more tumor-specific therapies with less severe side effects are needed. In the case of BL and *MYCN*-amplified NB, drugs that specifically target cells with deregulated MYC may provide such a treatment.

MYC overexpression has been shown to sensitize cells to apoptosis in response to a variety of cellular stresses, including death receptor ligation, hypoxia, DNA damage and cytotoxic drugs. The pro-apoptotic role of MYC overexpression is mainly exhibited when survival factors are limiting. Several apoptosis signaling pathways such as the p14/ARF-p53 pathway are activated by MYC overexpression. In addition, there is a pro-apoptotic shift in the balance among the Bcl-2 family proteins (reviewed in [17]). The pro-apoptotic function of overexpressed MYC is often overcome during tumor development by various additional lesions that block apoptosis. These anti-apoptotic changes may also contribute to drug resistance [18]. Hence, the role of MYC in drug responses is not clarified and there are conflicting data regarding the effect of MYC overexpression on the sensitivity to cytotoxic agents [19]–[29]. Identification of pathways that MYC overexpressing cancer cells, but not normal cells, depend upon for their survival is an attractive strategy for understanding the best way of treating MYC-deregulated cancers. Here we used a library of 80 cytotoxic compounds with defined mechanisms of action in order to identify drugs, which are more potent in cells that overexpress MYC compared to those with normal levels. Using the human B-cell derived p493-6 and the human NB-derived Tet21 N cell lines with conditional expression of c-MYC and MYCN respectively, we show that a limited number of agents, acting through distinct mechanisms, are more effective in MYC overexpressing tumor cells.

## Results

### MYC overexpression enhances the effect of a limited set of conventional cytotoxic drugs

To analyze the impact of deregulated MYC expression on the effect of conventional cytotoxic drugs with known mechanisms of action, we screened a library of 80 selected compounds used in the clinic or for research purposes (Table S1). The amount of viable cells after 48 h treatment using 1 µM of each drug was evaluated in order to identify drugs, which resulted in fewer viable cells in MYCN overexpressing Tet21 N cells or in c-MYC overexpressing p493-6 cells as compared to cells in which MYC was turned off. Using a difference of 30% as cut-off, 15 drugs scored positive in both cell lines (Table 1). In addition, four drugs (actinomycin D, cycloheximide, geldanamycin and 17-allylamino-geldanamycin) scored positive only in the MYCN overexpressing cells whereas aphidicolin was the single drug that was more effective only in the c-MYC overexpressing cells. In summary, there was a large overlap of positive hits between the two cellular systems used, indicating that overexpression of either c-MYC or MYCN in different cellular contexts results in similar response to treatment with cytotoxic drugs.

**Table 1:** Drugs that selectively targets MYC overexpressing cells.

SUBSTANCE	TARGET/EFFECT	ASSAY*	
		Tet21 N (MYCN)	p493-6 (c-MYC)
***Potentiation by MYCN and c-MYC***			
Mitomycin C	alkylating agent	**++**	**+++**
Methotrexate	dihydrofolate reductase (DHFR) inhibitor (inhibition of DNA and RNA synthesis)	**++**	**++**
Trichostatin-A	histone deacetylase inhibitor	**++**	**+++**
Anisomycin	protein synthesis inhibitor	**++**	**++**
Staurosporine	kinase inhibitor	**+++**	**++**
Gliotoxin	inhibitor of 20 S-proteasome chymotrypsin activity	**++**	**++**
MG-132	proteasome inhibitor	**+++**	**++**
Camptothecin	topoisomerase I inhibitor	**+++**	**++**
10-Hydroxycamptothecin	topoisomerase I inhibitor	**+++**	**++**
Daunorubicin	DNA intercalator. Inhibits topoisomerase I and II. RNA and DNA synthesis inhibitor. Induces DNA single-strand breaks	**+++**	**++**
Doxorubicin	DNA intercalator Inhibits topoisomerase I and II. RNA and DNA synthesis inhibitor. Induces DNA single-strand breaks	**+++**	**++**
Etoposide	topoisomerase II poison	**++**	**++**
Podophyllotoxin	tubulin polymerization inhibitor	**+++**	**++**
Paclitaxel	microtubule stabilizer	**+++**	**++**
Vinblastine	tubulin inhibitor	**+++**	**+++**
***Potentiation by MYCN***			
Actinomycin D	RNA synthesis inhibitor	**+++**	NE
Cycloheximide	protein synthesis inhibitor	**++**	+
17-Allylamino-geldanamycin	HSP-90 inhibitor	**+++**	+
Geldanamycin	HSP90 inhibitor	**+++**	+
***Potentiation by c-MYC***			
Aphidicolin	DNA polymerase inhibitor (inhibition of DNA synthesis)	**+**	+++

*Decrease in number of viable cells expressed as % inhibition in MYC-ON cells compared to MYC-OFF cells, calculated as the difference between (optical density [OD] for drug treated MYC-OFF cells)/(OD for DMSO treated MYC-OFF cells) and (OD for drug treated MYC-ON cells)/(OD for DMSO treated MYC-ON cells).

+, 10–35% inhibition; ++, 35–50% inhibition; +++,≥50% inhibition; NE  =  No effect (≤10% effect).

**Figure 1:**
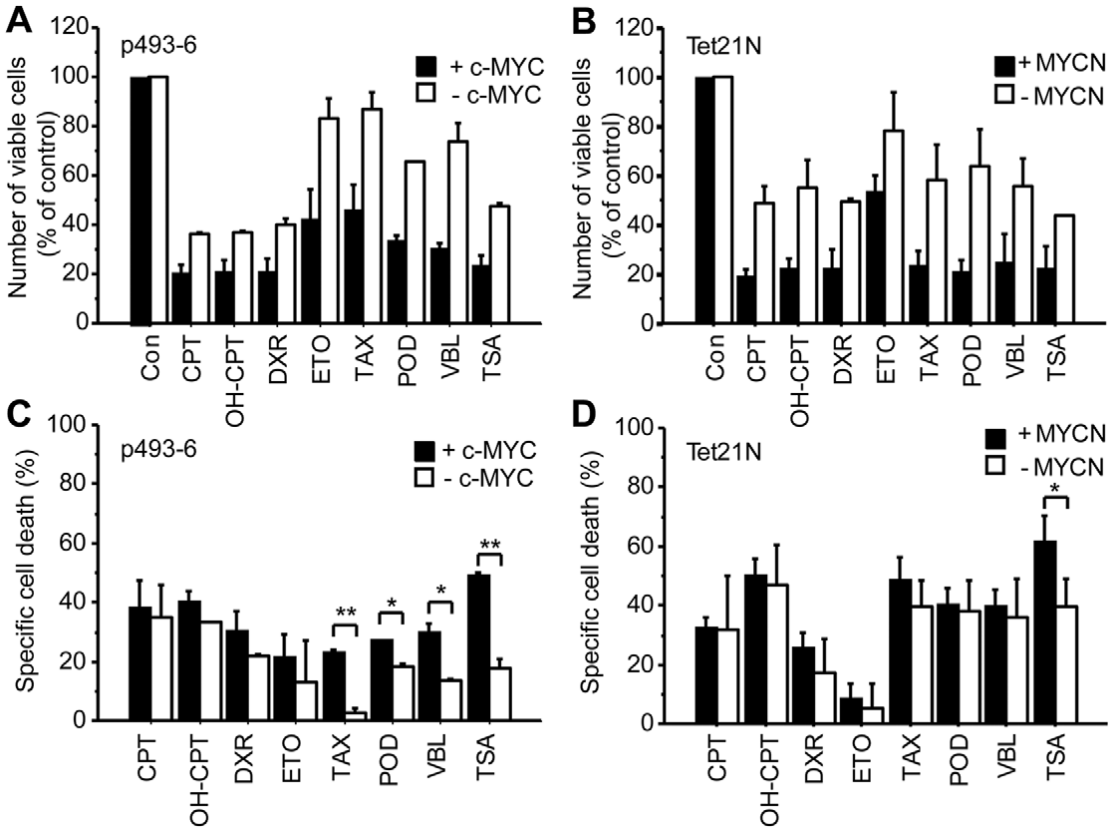
Effects on cell proliferation and cell death by selected MYC specific compounds. Quantification of the amount of viable cells after 48 h of treatment with 1 µM of the indicated drugs, using WST-1 assay in p493-6 cells (**A**) or crystal violet staining in Tet21 N cells (**B**). Cells were cultured in the presence of doxycycline (1 µg/ml) to turn off MYC expression or without doxycycline to allow MYC overexpression. Data are shown as percent of control (DMSO) treated cells and represent the means of two independent experiments. Error bars indicate standard deviation. Quantification of cell death by propidium iodide staining for sub-G1 DNA content of p493-6 cells (**C**) and Tet21 N cells (**D**), treated as in (**A**) and (**B**). Data are shown as drug specific cell death induction (see Material and Methods) relative to control treated cells and represent the means of at least three independent experiments. Error bars indicate standard deviation. * p<0.01, ** p<0.0001.

Although the drugs showing potentiated effect by MYC overexpression acted on different cellular targets, there was enrichment for compounds belonging to certain drug classes. Not surprisingly many drugs targeting cycling cells were potentiated by MYC overexpression. All three microtubule-targeting agents included in the library had a greater effect when either c-MYC or MYCN was overexpressed. In addition, 5 out of 7 topoisomerase inhibitors were more than 30% more efficient when either MYCN or c-MYC was overexpressed (Figure 1A, B, Table 1, Table S1). The remaining two topoisomerase II inhibitors, Ellipticine and ICRF-193, showed some potentiation by MYC overexpression but failed to reach our cut-off (Table S1). Furthermore, both proteasome inhibitors included were selected by the screen, as were both protein synthesis inhibitors, although the effect of cycloheximide was only potentiated in the MYCN overexpressing cells. In addition, drugs inhibiting DNA and RNA synthesis were selected. The effect of the two HSP90 inhibitors analyzed were both potentiated in the MYCN overexpressing Tet21 N cells only (Table 1 and Table S1). In conclusion, our data suggested that MYC overexpression potentiated the effect of drugs acting through several different, yet specific sets of mechanisms.

### Effect of MYC overexpression on apoptosis induced by the cytotoxic compounds

Since there seemed to be selectivity in the mechanisms of the drugs whose effect could be potentiated by MYC overexpression, we chose to further investigate the effect of four of the topoisomerase inhibitors (Camptothecin [CPT], 10-hydroxy-camptothecin [OH-CPT], Etoposide [ETO] and Doxorubicin [DXR]) and the three microtubule-targeting agents (Paclitaxel [TAX], Podophyllotoxin [POD] and Vinblastine [VBL]). The histone deacetylase inhibitor Trichostatin-A (TSA) was also included (Figure S1). To validate the MYC dependent effect we determined the concentration dependence and IC50 values of these drugs in the two screening assays used. All drugs displayed a concentration dependent effect over the range of concentrations examined (0.5 nM to 15 µM), except for TAX in p493-6 cells (Table 2, Figure S2). The IC50 values for the four topoisomerase inhibitors and the HDAC inhibitor TSA were all statistically significantly lower in c-MYC/MYCN overexpressing cells (p<0.05) compared to the low expressing counterparts. The three microtubule-targeting drugs also showed lower IC50 values in the MYC overexpressing cells but this did in most cases not reach a statistical significance (Table 2). Thus, MYC overexpression enhanced the response to the majority of the selected drugs over a broad range of concentrations.

**Table 2:** IC50 values in cells with low versus high MYC-expression status.

	Tet21 N		P493-6	
	MYCN on	MYCN off	c-MYC on	c-MYC off
Drug	IC50 (95% CI*)	IC50 (95% CI)	IC50 (95% CI)	IC50 (95% CI)
Camptothecin	5.4 nM (3.5–7.3)^a^	73 nM (48–97)	5.6 nM (2.4–8.7) a	49 nM (28–70)
10-hydroxy-camptothecin	11 nM (7.2–15) a	74 nM (40–110)	4.2 nM (1.0–7.4) a	72 nM (31–110)
Etoposide	1.5 µM (0.85–2.2) a	>15 µM	0.22 µM (0.07–0.38)^a^	>15 µM
Doxorubicn	98 nM (69–130) a	400 nM (220–580)	26 nM (5.8–45) a	250 nM (150–340)
Podophyllotoxin	3.9 nM (2.8–5.0)	6.6 nM (4.1–9.0)	6.0 nM (3.0–9.0)	20 nM (3.5–37)
Paclitaxel	4.1 nM (2.0–6.2)	30 (<0.5–63)	-	-
Vinblastine	1.1 nM (0.6–1.6) a	28 (3.8–52)	<0.5 nM	36 nM (<0.5–73)
Trichostatin-A	62 nM (50–75) a	130 (88–1200)	17 nM (6.6–27) a	140 nM (77–200)

*CI; Confidence interval.

a Statistically significant difference at the 95% confidence-level in the IC50 values between MYC on and MYC off conditions.

The crystal violet and WST-1 assays used in the screen measure the combined effect of the drugs on cell growth inhibition and cell death (Figure 1A, B). Since MYC promotes proliferation and MYC overexpression can sensitize cell to apoptosis, the influence of MYC expression on the outcome of these drugs could be due to effects on either of these processes. To determine the effect of MYC overexpression on the induction of apoptosis, we quantified cells with subG1 DNA content in drug-treated p493-6 and Tet21 N cells in the presence or absence of doxycycline (Figure 1C, D). In p493-6 cells, TSA and the three microtubule-targeting agents (TAX, POD VBL) induced significantly more apoptosis when c-MYC was overexpressed. The three topoisomerase inhibitors (10-OH-CPT, DXR and ETO) showed a similar tendency but this failed to reach statistical significance. In the Tet21 N cells on the other hand, even though all drugs induced apoptosis to variable extent, only TSA induced significantly more apoptosis in the MYCN overexpression setting. This indicated that the difference seen in the crystal violet assay depending on MYCN status (Figure 1B) at this drug concentration was due to effects on proliferation, or alternatively due to modes of cell death not involving DNA fragmentation.

**Figure 2:**
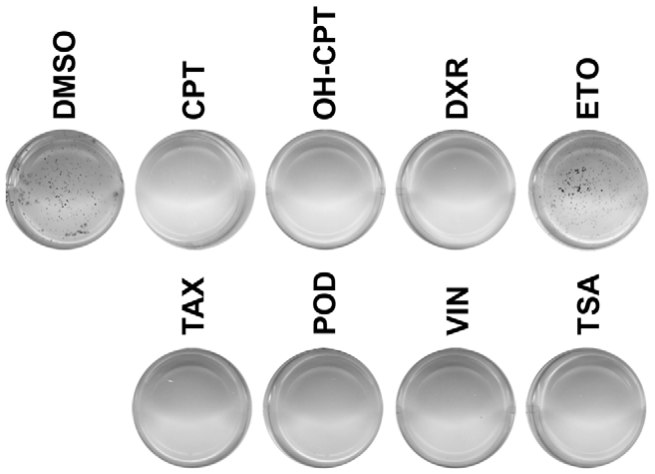
Inhibition of anchorage independent growth of neuroblastoma cells by selected MYC-specific compounds. SK-N-BE(2) cells were seeded in soft agar and treated with 1 µM of the eight reference compounds indicated. For each compound, the colonies were counted and representative plates from two independent experiments performed in duplicates are presented. DMSO treated cells were used as control.

The p53-deficient *MYCN*-amplified NB cell line SK-N-BE(2) efficiently forms colonies when plated in soft agar. All drugs, with the exception of ETO, completely inhibited anchorage independent growth in soft agar of these cells at a concentration of 1 µM (Figure 2). Increasing the ETO concentration to 10 µM also resulted in complete inhibition of colony formation (data not shown). This shows that the selected drugs could target *MYCN*-amplified neuroblastoma, independently of p53 activation.

### Inhibition of topoisomerase I decreases c-MYC expression levels

**Figure 3:**
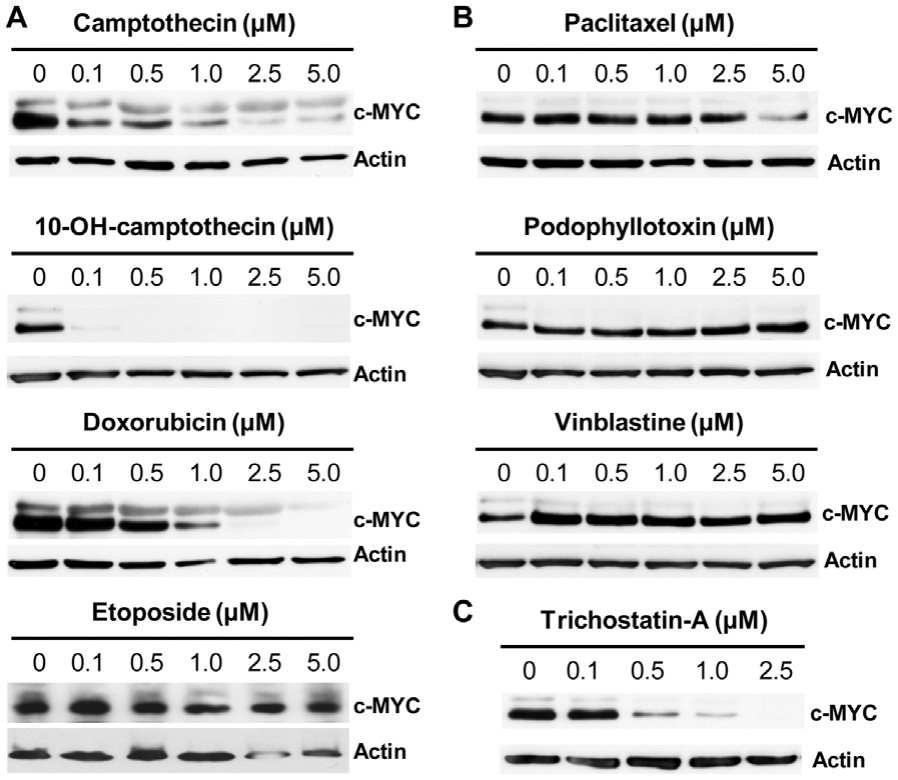
Effects of drug treatment on c-MYC protein levels. (**A**) Western blot analysis of c-MYC expression in p493-6 cells treated for 48 h with indicated concentrations of topoisomerase inhibitors (**A**), microtubule-targeting agents (**B**) or the HDAC-inhibitor trichostatin-A (**C**). Membranes were probed with antibodies recognizing c-Myc and Actin was used as a loading control.

**Figure 4:**
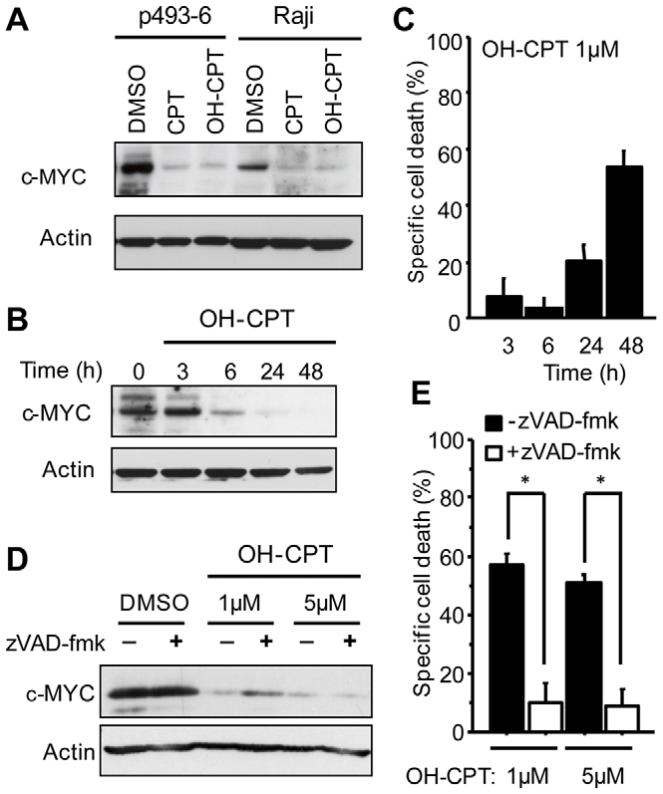
c-MYC down-regulation upon topoisomerase I inhibition precedes cell death induction. (**A**) Western blot of c-MYC expression in p493-6 and Raji cells after 48 h treatment with 1 µM of the indicated drugs. (**B**) Western blot analysis of c-MYC expression in p493-6 cells before and after treatment for indicated times with 1 µM of 10-OH-Camptothecin. (**C**) Cell death induction as measured by propidium iodide staining and quantification of cells with subG1 DNA content after treatment for indicated times with 1 µM of 10-OH-Camptothecin. Data are shown as specific cell death induction relative to control (DMSO) treated cells and represent the means of three independent experiments. Error bars indicate standard deviation. (**D**) Western blot analysis c-MYC expression in p493-6 treated for 48 h with topoisomerase inhibitors in the presence or absence of the pan-caspase inhibitor zVAD-fmk. (**E**) Cell death induction as measured by propidium iodide staining and quantification of cells with subG1 DNA content of cells treated with topoisomerase inhibitors in the presence or absence of the pan-caspase inhibitor zVAD-fmk for 48 h. Data are shown as specific cell death induction relative to control (DMSO) treated cells and represent means from three independent experiments. Error bars indicate standard deviations. * p<0.01.

Experimental models have shown that some tumors can be dependent on sustained expression of MYC, referred to as oncogene addiction. Hence withdrawal of MYC can lead to tumor regression associated with tumor cell death, senescence and/or differentiation [30]–[32]. With this in mind we wanted to understand if the enhanced efficacy of some of the compounds observed in MYC overexpressing cells could be due to inhibition of MYC function. We therefore monitored the effects of drug treatment on c-MYC protein expression. Western blot analysis of c-MYC revealed a decrease in the c-MYC protein levels after treatment with the topoisomerase I inhibitors CPT and OH-CPT, as well as with the HDAC inhibitor TSA and to a lesser extent DXR, which can inhibit both topoisomerase I and II (Figure 3A, C). In contrast, we could not observe any significant effect on c-MYC protein levels after treatment with ETO or with any of the microtubule-targeting agents. If anything, there was a slight increase in protein levels (Figure 4A, B).

Since c-MYC is expressed from a Tet-responsive promoter in the p493-6 cells the effect of the drugs on c-MYC expression levels could be due to unspecific effect on the Tet-OFF-system. To rule this out we analyzed whether any of the drugs affected the luciferase signal in a cell line expressing the firefly luciferase gene under the control of a Tet-responsive promoter (CHO AA8-Luc Tet-OFF). Doxycyline-treatment led, as expected, to a robust decrease in the luciferase activity (Figure S3). In contrast, we could not detect any decrease in the luciferase signal after treatment with 1 µM of any of the cytotoxic drugs tested, verifying that they did not mimic the effect of doxycycline in the Tet-OFF system (Figure S3). In addition we treated the BL cell lines Raji and Daudi with CPT and OH-CPT to analyze effects on MYC expression. Treatment with 1 µM of each drug for 48 h resulted in a similar decrease in c-MYC levels in these BL cell lines as in the p493-6 cells (Figure 4A and data not shown). Together these experiments show that the observed down-regulation of c-MYC in the p493-6 was not due to unspecific effects on the Tet-OFF system but worked through an alternative mechanism.

To validate that the decreased c-MYC levels after topoisomerase I inhibition was not simply due to cell death we monitored kinetics of c-MYC regulation and cell death induction after treatment of p493-6 cells with OH-CPT. Upon treatment with 1 µM of the topoisomerase I inhibitor c-MYC down-regulation was evident already within 6 h (Figure 4A), while apoptosis induction was observed at low levels after 24 h and was fully evident only after 48 h (Figure 4B). Furthermore, treatment with the caspase inhibitor zVAD-fmk, which almost completely blocked OH-CPT-induced apoptosis, could not rescue the c-MYC protein levels (Figure 4C, D). In summary, our data indicate that inhibition of topoisomerase I leads to a rapid reduction of c-MYC levels that precedes the induction of apoptosis.

### MYC/Max binding to DNA is disrupted by DXR

**Figure 5:**
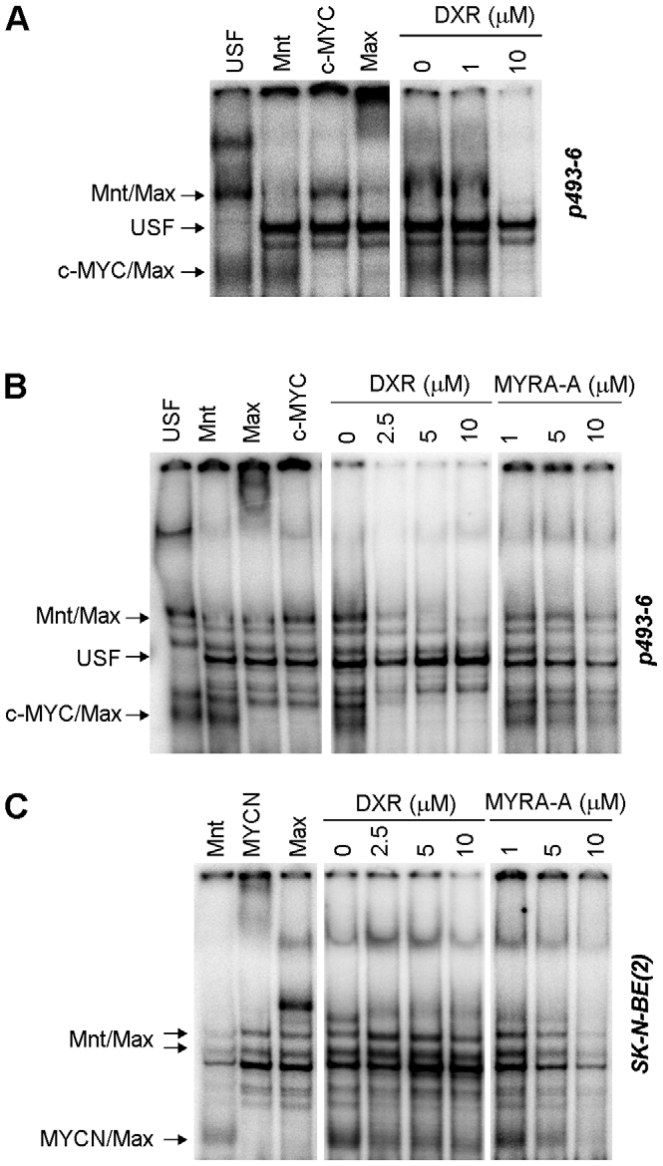
Doxorubicin inhibits MYC/Max DNA binding. Electromobility shift assay showing binding of MYC network proteins in extracts from p493-6 cell treated *in vitro* with DXR (**A**), or extracts from p493-6 cells (**B**) or SK-N-BE(2) cells (**C**) treated in culture for 48 h with DXR or MYRA-A. The Mnt/Max, USF, c-MYC/Max, and MYCN/Max DNA–protein complexes were identified by antibody super shifts and are indicated to the left.

To further explore effects on MYC function, we performed electrophoretic mobility shift assays (EMSAs) on cell extracts treated with the different drugs to evaluate their effect on DNA binding of the c-MYC/Max complex. At drug concentrations up to 100-fold higher than those in the original screen, seven of the eight tested drugs had virtually no effect on the amount of c-MYC/Max present on the E-box-containing CMD oligonucleotide when incubated with p493-6 extracts (data not shown). In contrast, DXR, completely disrupted DNA binding of complexes containing c-MYC, Max or Mnt already at 10 µM, while USF binding was apparently not affected (Figure 5A).

To further corroborate this finding, we also performed EMSAs on extracts from cells that had been treated with DXR in culture, as well as on extracts from cells treated with MYC Pathway Response Agent-A (MYRA-A), a MYC pathway specific apoptosis inducer that we previously showed to disrupt binding of MYC/Max to DNA [33], [34]. c-MYC/Max DNA binding was abrogated in treated p493-6 cells at the lowest tested concentrations of the drugs (2.5 µM and 5 µM respectively, Figure 5B). Similar results were seen for MYCN/Max binding in DXR and MYRA-A treated SK-N-BE(2) cells (Figure 5C). In summary, our data show that DXR and MYRA-A can interfere with the DNA binding of c-MYC/Max as well as MYCN/Max, and thus likely influences the transcriptional activity of these complexes.

## Discussion

MYC oncoproteins are aberrantly expressed in a wide variety of human cancers, in most cases as a result from mutations at one or multiple levels of the MYC pathway, making them attractive targets for cancer therapy [1], [9]. Several studies of tumor formation in *in vitro* and *in vivo* systems with conditional MYC expression have demonstrated that MYC induction promotes tumor formation while its down-regulation causes tumor cell growth arrest, enhanced apoptosis, senescence, tumor regression and/or differentiation (for review see [35]). Animal studies have shown that even a transient MYC downregulation appears to be sufficient for diminishing tumor tissue and that the tumor may actually regress upon MYC reactivation [30], [31], [36], [37]. Furthermore, it was recently demonstrated that systemic inhibition of endogenous MYC activity in mice caused regression of Ras-dependent tumors while the effects on normal regenerating tissues were well tolerated and completely reversible [32]. Thus, interfering with the MYC pathway would potentially enable elimination of malignant cells with low side effects for normal cells. Alternatively, inhibition of pathways that MYC overexpressing cells depend on for their survival could be exploited for targeting these tumors.

Here, we have explored how substances with already characterized cellular targets affect the MYC pathway and demonstrated that the effects of only a subset of drugs, acting on selected cellular targets, were enhanced by MYC overexpression. Contradictory results regarding the correlation between MYC expression levels and cellular sensitivity to chemotherapeutic drugs exist in the literature. Reports showing that c-MYC overexpression confers drug resistance [19], [20], [28] are challenged by others, including studies from our group, demonstrating MYC-mediated sensitization to a number of chemotherapeutic drugs [21], [24]–[27]. This may be due to diverse effects elicited by various drugs or by different cellular contexts. We show in this study that the cytotoxic effects of only a subset of the drugs in our screen were potentiated by MYC overexpression, indicating that overexpression of MYC in cancer cells does not lead to a general sensitization to cytotoxic drugs. Instead our results suggest that MYC overexpression makes cells vulnerable to targeting of specific biological processes. Particularly, drugs targeting microtubules and topoisomerases were identified. All three microtubule-targeting agents included in the screen induced significantly more apoptosis in MYC overexpressing cells. These agents act by disrupting spindle microtubule dynamics during the mitotic phase of the cell cycle, leading to activation of the spindle assembly checkpoint and mitotic arrest followed by cell death [38]. Our data is in line with the observations by others that MYC sensitized cells to inhibition of Aurora kinases and CDK1 [39]–[41] suggesting that MYC overexpressing cells are particularly sensitive to disruption of these mitotic processes.

It has been suggested that the response to treatment is dependent on which member of the MYC family that is deregulated [42]. However, we observed to a large extent, similar effects on cytotoxicity in cells with either deregulated c-MYC or MYCN, indicating similar biological effects on the cells. This is in agreement with recent demonstrations that high MYC-pathway activity is associated with poor outcome of the disease in neuroblastoma patients, irrespective of whether it was caused by *MYCN*-amplification, by increased *c-MYC* level or by any other cause [43], [44].

We observed that the topoisomerase I inhibitors, as well as the histone deacetylase inhibitor TSA, led to down-regulation of MYC protein levels. Decreased MYC levels were also recently reported after treatment with the topoisomerase I inhibitor Sn38 [45]. The mechanism behind this down-regulation is currently unresolved. In contrast we did not observe any down-regulation of MYC levels by microtubule-targeting agents, and the relationship between MYC expression and the cytotoxic effect of these agents appears to be largely context dependent [23], [26]–[28], [46], [47].

Doxorubicin was found to be unique among the studied drugs since it could disrupt the binding of the MYC/Max complex to DNA. We have previously shown a similar effect by the small molecule MYRA-A [33]. Interestingly, the structure of MYRA-A shares some similarity with that of DXR (Figure S1), suggesting that they may act in an analogous way. DNA binding properties of MYRA-A has indeed been suggested by others [48]. Similar to what was previously shown for MYRA-A [33], DXR also interfered with binding of Mnt/Max complexes to DNA, while binding of USF remained unaltered. Hence not all DNA binding proteins were affected. Even though USF binds to the same E-box sequences as MYC/Max this could indicate different modes of DNA-binding for the different factors. Detailed structural studies would be required to understand how DXR and MYRA-A affect the binding of these proteins to DNA.

In conclusion, overexpression of MYC renders tumor cells particularly sensitive to targeting of certain mechanisms and pathways, including topoisomerases and the mitotic control machinery as shown in this study. Discovery of novel substances is essential for development of future cancer therapies. It is also important to analyze how the conventional cytotoxic drugs used in the clinic today act in different cellular contexts. Comparison between cells with normal and overexpressed MYC will help to enhance our understanding of how to best use and combine conventional drugs in cancer treatment. This information will hopefully also be useful when developing novel cancer therapies.

## Materials and Methods

### Cell culture and drug treatments

The human NB SH-EP cell line with conditional MYCN expression (Tet- 21 N, Tet-OFF system [49] was cultured in RPMI 1640 medium supplemented with 10% FBS, 1% glutamine, 1% penicillin/streptomycin, G418 (200 µg/ml), and hygromycin (100 µg/ml). The human B-cell line p493-6 with tetracycline regulated (Tet-OFF) c-MYC expression [50] and the BL cell lines Raji and Daudi was cultured in RPMI 1640 medium supplemented with 10% FBS, 1% glutamine, and 1% penicillin/streptomycin. MYCN and c-MYC expression was regulated by addition of doxycycline (l µg/ml, Sigma) for 20 h before drug treatment. Human NB SK-N-BE(2) cells with amplified *MYCN* were a gift from Per Kogner (Karolinska Institutet, Sweden) and were grown in EMEM:F12-Ham medium (1∶1, v/v) supplemented with 10% FBS, 1% glutamine, 1% non-essential amino acids, and 1% penicillin/streptomycin. The Chinese hamster ovary derived CHO AA8-Luc Tet-OFF cell line (Clontech), was maintained in EMEM supplemented with 10% Tet-system approved FBS, 1% L-glutamine, and 1% penicillin/streptomycin.

### Reagents

The chemical library provided by Actar AB (Stockholm, Sweden), consisted of 80 cytotoxic reference compounds with known cellular targets (Table S1). Propidium Iodide and RNase A were from Sigma and zVAD-fmk was from AG Scientific Inc. (San Diego, CA). MYRA-A was kindly provided by NCI (Bethesda MD).

### Cell viability assays

To quantify the amount of living cells the WST-1 Cell Proliferation reagent (Roche Diagnostics) was used for the non-adherent p493-6 cells, according to the manufacturer's instructions, while adherent Tet21 N cells were stained with crystal violet (Sigma) as previously described [33].

### IC50 determination

Tet-21 N and p493-6 cell were treated with drugs in a concentration ranging from 0.5 nm to 15 µM for 48 h in the presence or absence of doxycycline, after which the amount of viable cells were quantified. For calculation of IC50 values with 95% confidence intervals Four Parameter Logistic best-fit concentration-response curves were generated using XLfit add-in for Excel (IDBS). Statistical significance for the differences in IC50 values was determined based on the 95% confidence intervals.

### Flow cytometry analysis

Drug-induced apoptosis was scored by quantifying the subG1 fraction. Cells were fixed with 70% ice-cold methanol and stored at +4°C over night or −20°C for longer times. Cells were stained with propidium iodide solution (5 µg/ml propidium iodide and 25 µg/ml RNase A in PBS) for 30 min at 37°C, and analyzed in a FACScan flow cytometer (Becton Dickinson). Apoptotic cells were identified within the PI-stained population by virtue of exhibiting an apparent sub diploid DNA content. Drug specific cell death was defined as the percentage of apoptosis induced when adjusted for background cell death level in control treated cells and calculated as [(% apoptosis in drug treated cells)-(% apoptosis in control treated cells)]/[100%-(% apoptosis in control treated cells)]*100%.

#### Luciferase Assay

Luciferase activity was measured using the Dual-Luciferase Reporter Assay (Promega) according to the instructions from the manufacturer. Protein concentrations of the extracts used were determined using the Bradford method (Bio-Rad Protein Assay, Bio-Rad). Luciferase signals were normalized to the protein concentration.

### Immunobotting

Whole-cell extracts were prepared using F-buffer and Western blot analysis was performed as previously described [25]. Membranes were probed with mouse monoclonal anti-c-MYC antibody (9E10, Santa Cruz Biothecnology Inc). Equal loading of proteins was confirmed by re-probing the membranes with antibodies specific for Actin (Sigma). HRP-conjugated anti-mouse antibody (Amersham Biosciences) served as secondary reagent. Blots were developed by enhanced chemiluminescence (ECL, Amersham).

### Electrophoretic mobility shift assay (EMSA)

To assess the drug effect on DNA binding of the MYC/Max complex, EMSA was performed essentially as previously described [51], using protein extracts from cells treated either in culture (48 h incubation) or protein extracts treated *in vitro* with drug. Briefly, 15 µg of protein from SK-N-BE(2) or p493-6 cells was incubated for 30 min with sonicated salmon sperm DNA, antibodies to identify relevant bands by super shift, and drugs for the in vitro experiments at 25°C. This initial incubation was followed by 30 min incubation with [γ32P]ATP end-labeled CMD probe at 25°C. The DNA-protein complexes were then separated on 5.5% polyacrylamide gels in 25 mM Tris base, 20 mM boric acid, and 0.5 mM EDTA at 4°C, dried and visualized on a phosphoimager. Specific bands were identified by using the following antibodies for super shifts: M132 (Mnt), X (USF), N262 (c-MYC), NCMII (MYCN) and C-17 (Max); all from Santa Cruz Biotechnology, Inc.

### Soft agar experiments

Single-cell suspension of SK-N-BE(2) cells in 0.4% SeaPlaque agarose (Cambrex BioScience-Rockland, East Rutherford, NJ) was seeded onto a 0.6% agarose base. After one hour, medium containing the selected reference compounds was added to the top of the gel and cultures were then incubated for 4 days. Medium was thereafter changed every 3–4 days. Following incubation for approximately 2 weeks, living cells and colonies were counted and visualized by the addition of 100 µg/ml MTT reagent (Calbiochem). Two independent experiments were performed in duplicates and the number of colonies formed in drug treated cells was compared to the control (DMSO).

## Supporting Information

Figure S1 **Structures of the compounds used in the study.** Structures for the eight cytotoxic drugs selected for further characterization of their effect on the MYC-pathway as well as the structure of MYRA-A.
(TIF)

Figure S2 **Concentration response curves for IC50 calculations. (A)** Concentration-response curves for p493-6 cells treated with the indicated drugs in the presence (c-MYC OFF) or absence (c-MYC ON) of doxycycline for 48 h. The amount of viable cells was quantified using the WST1 reagent and the percentage of viable cells relative to control (DMSO) treated cells are depicted. **(B)** Concentration-response curves for Tet21 N cells treated with the indicated drugs in the presence (MYCN OFF) or absence (MYCN ON) of doxycycline for 48 h. The amount of viable cells was quantified by crystal violet staining and the percentage of viable cells relative to control (DMSO) treated cells is depicted. Data represent 3–5 independent experiments.
(TIF)

Figure S3 **Cytotoxic drugs do not mimic the effect of doxycycline on the Tet-OFF promoter.** CHO-AA8 Tet-OFF-Luc cells were treated for 48 h with 1 µM of the indicated cytotoxic drugs or with 1 µg/ml doxycycline. Luciferase activity was assayed with the Dual-Luciferase Reporter Assay and the luciferase signal was normalized to the protein concentration in each extract analyzed. Data represent triplicates in each experiment and error bars indicate standard deviation.
(TIF)

Table S1 **Characteristics of the reference substances used in the study.** List of the 80 compounds in the chemical library and their cellular targets. The effect on viability in cells overexpressing MYCN or c-MYC relative to non-overexpressing cells is shown for each compound.
(XLS)
