# Supplementary figures and images for: Identification of Cytotoxic Drugs That Selectively Target Tumor Cells with MYC Overexpression
Source: PLoS One. 2011 Nov 23;6(11):e27988. doi: 10.1371/journal.pone.0027988 (PMC3223192; doi:10.1371/journal.pone.0027988)

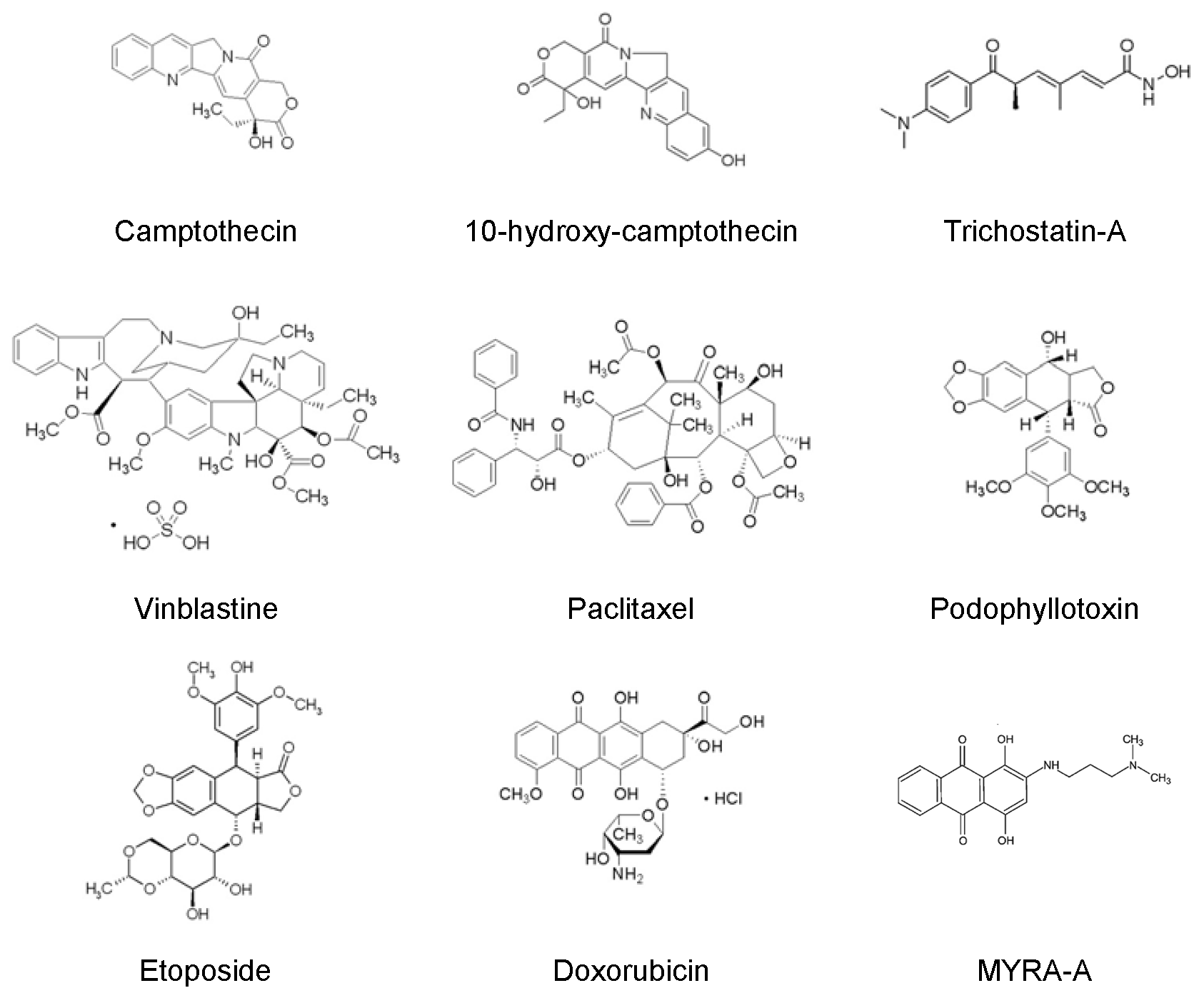

Supplement: Figure S1 — Structures of the compounds used in the study. Structures for the eight cytotoxic drugs selected for further characterization of their effect on the MYC-pathway as well as the structure of MYRA-A. (TIF) [file pone.0027988.s001.tif]

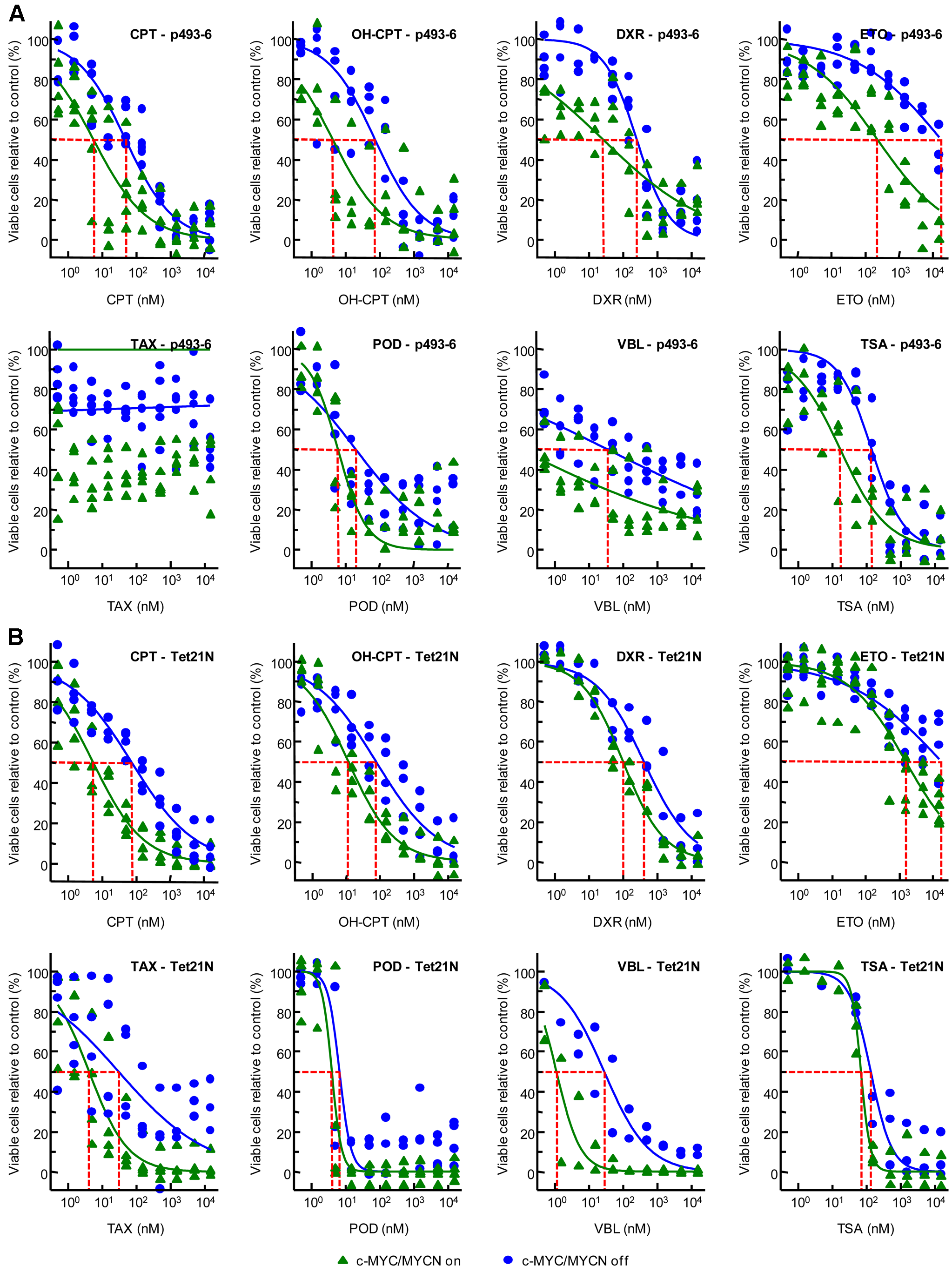

Supplement: Figure S2 — Concentration response curves for IC50 calculations. (A) Concentration-response curves for p493-6 cells treated with the indicated drugs in the presence (c-MYC OFF) or absence (c-MYC ON) of doxycycline for 48 h. The amount of viable cells was quantified using the WST1 reagent and the percentage of viable cells relative to control (DMSO) treated cells are depicted. (B) Concentration-response curves for Tet21 N cells treated with the indicated drugs in the presence (MYCN OFF) or absence (MYCN ON) of doxycycline for 48 h. The amount of viable cells was quantified by crystal violet staining and the percentage of viable cells relative to control (DMSO) treated cells is depicted. Data represent 3–5 independent experiments. (TIF) [file pone.0027988.s002.tif]

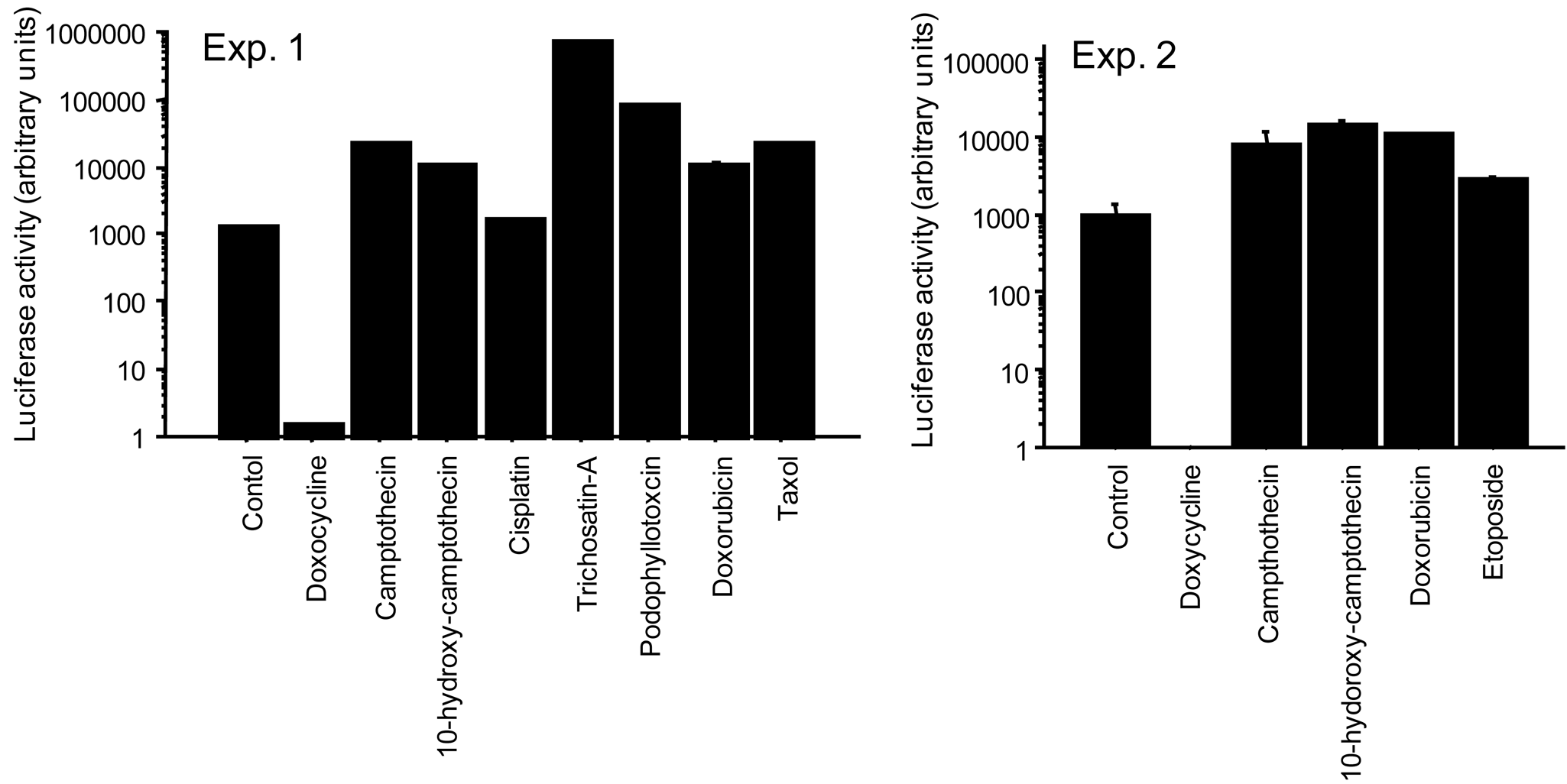

Supplement: Figure S3 — Cytotoxic drugs do not mimic the effect of doxycycline on the Tet-OFF promoter. CHO-AA8 Tet-OFF-Luc cells were treated for 48 h with 1 µM of the indicated cytotoxic drugs or with 1 µg/ml doxycycline. Luciferase activity was assayed with the Dual-Luciferase Reporter Assay and the luciferase signal was normalized to the protein concentration in each extract analyzed. Data represent triplicates in each experiment and error bars indicate standard deviation. (TIF) [file pone.0027988.s003.tif]
